# Effects of drip tape modes on soil hydrothermal conditions and cotton yield (*Gossypium hirsutum* L.) under machine-harvest patterns

**DOI:** 10.7717/peerj.12004

**Published:** 2021-08-23

**Authors:** Dongwang Wang, Zhenhua Wang, Tingbo Lv, Rui Zong, Yan Zhu, Jinzhu Zhang, Tianyu Wang

**Affiliations:** 1 Shihezi University, Key Laboratory of Modern Water-Saving Irrigation of Xinjiang Production and Construction Corps, Shihezhi, Xinjiang, China; 2 Shihezi University, College of Water Resources and Architectural Engineering, Shihezhi, Xinjiang, China

**Keywords:** Drip tape modes, Soil temperature, Soil moisture, Soil salinity, Cotton yield and quality, Water use efficiency, Machine-harvest cotton

## Abstract

**Background:**

The layout of drip tapes under mulch has changed in Xinjiang, China, with the development of machine-harvest cotton (*Gossypium hirsutum* L.) planting technology. This study aims to demonstrate the effects of drip tape modes on soil hydrothermal conditions, cotton yield, and water use efficiency (WUE) of machine-harvest cotton under mulch in Xinjiang.

**Methods:**

A field experiment was conducted to set up two machine-harvest cotton planting patterns (T1: the cotton planting model with one film, two drip tapes and six rows; T2: the cotton planting model with one film, three drip tapes and six rows), and a conventional planting mode (T3: the cotton planting model with one film, two drip tapes and four rows) as a control.

**Results:**

Our results showed that the heat preservation and warming effects of the cotton planting model with one film, two drip tapes and six rows and the cotton planting model with one film, three drip tapes and six rows were better than that of the conventional planting mode. Soil temperature under the mulching film quickly increased and slowly decreased, which was beneficial to the early growth and development of cotton. The mean soil moisture content of the 0–60 cm soil layer in the cotton planting model with one film, three drip tapes and six rows was significantly higher than the other two treatments at the middle and late stage of cotton growth (90 days after sowing (DAS) and 135 DAS). Moreover, the water holding capacity of the middle and upper part of the tillage layer in the cotton planting model with one film, three drip tapes and six rows was the best. At the medium cotton growth stage, the main root layer in the cotton planting model with one film, three drip tapes and six rows formed a desalination zone. At the late cotton growth stage, the soil salinity content of the 0–60 cm soil layer showed that the cotton planting model with one film, three drip tapes and six rows was the lowest, the cotton planting model with one film, two drip tapes and six rows was the highest, and the conventional planting pattern was in the middle. Among these three modes, the cotton planting model with one film, three drip tapes and six rows was more efficient in controlling soil salt accumulation. The agronomic traits and cotton quality in the cotton planting model with one film, three drip tapes and six rows were better than that for the other two treatments. Compared with the other treatments, the cotton yield in the cotton planting model with one film, three drip tapes and six rows increased by 6.15% and 11.0% and 8.1% and 12.3%, in 2017 and 2018, respectively, and WUE increased by 17.4% and 22.7% and 20.9% and 22.8%, in 2017 and 2018 respectively. In conclusion, the cotton planting model with one film, three drip tapes and six rows can be recommended for machine-harvest cotton planting for arid areas in Xinjiang, considering water conservation and improving cotton yield.

## Introduction

Xinjiang is the largest cotton (*Gossypium hirsutum* L.) planting region in China. Abundant light and heat resources provide advantages for industrial cotton growing (Li & Li, 2014). The region, located in northwest China, includes a large area in which the high-quality cultivation technique of “dry sowing and wet out, dwarfing and close planting, and drip irrigation under mulch” is applied as it suits the local light and temperature conditions (Han & Yang, 2013; ICR, CAAS (Institute of Cotton Research, Chinese Academy of Agricultural Sciences), 2013). However, the shortage of water has become an important factor limiting agricultural development in Xinjiang (Liu et al., 2012a; Danierhan et al., 2013). To ensure the sustainable development of agriculture in this region by improving irrigation efficiency and controlling secondary salinization, surface drip irrigation under mulch films (*i.e*., mulched drip irrigation) has been in place since the 1990s (Liu et al., 2012b; Zhong et al., 2016). Drip irrigation under mulch film is the integration of drip irrigation technology and plastic film mulching cotton planting technology (Wang, Fan & Guo, 2019). Fertilizer can be dissolved in the water used for irrigation, so as to improve the efficiency of water and fertilizer use. Recently, with the application of drip irrigation technology and the improvement of industrial and agricultural production levels, the planting and harvesting of cotton is also developing in the direction of mechanization. The development of mechanization reduces production costs and labor intensity, improves the harvest efficiency, and improves the economic benefits of cotton (Wang et al., 2019; Ning, Zuo & Shi, 2013). The current conventional planting mode adopted by Xinjiang is the wide-row spacing pattern (30 cm + 60 cm). However, the conventional planting mode is not conducive to mechanized operation. First of all, the traditional cotton planting mode leads to higher levels of trash in the machine-picked seed cotton, which increases the ginning and processing steps. Increasing these steps causes poorer fiber quality in terms of fiber length, fiber strength, and short fiber proportion (Zhang, 2013; Abdazimov, Radjabov & Omonov, 2019). Moreover, insufficient market competitiveness results in major economic losses. Second, there are fewer cotton-picking machines suitable for this conventional mode, mainly manual picking, which increases costs and reduces cotton-picking efficiency. On the other hand, water and salt distribution are disadvantageous for cotton growth because of the arrangement of drip tapes under the conventional pattern (Wang et al., 2018a; Liu et al., 2012a).

To improve the quality and yield of machine harvested cotton and adapt to the mechanical harvest of cotton, the cotton planting mode and film width have changed greatly compared with those used in the traditional planting mode (Fan et al., 2013; Li et al., 2015). Although the conventional planting pattern adopted by Xinjiang is the wide-row spacing pattern (30 cm + 60 cm), the machine-harvested cotton planting pattern is the wide-narrow pattern (66 cm + 10 cm). The layout of drip tape has changed from the traditional four rows of cotton controlled by one film two drip tapes to one film three drip tapes controlling six rows of cotton. Moreover, the corresponding width of the covering film has changed from the traditional 1.45 m to 2.05 m, and the planting density of cotton has also changed (Bednarz et al., 2005; Wang et al., 2018a). Studies have shown that high or low density planting can significantly reduce yield (Zhang et al., 2016). Therefore, in the optimization of mechanized cotton planting, adjusting the layout of the drip tape and the planting density can improve the hydrothermal and light conditions of the group and subsequently have a positive impact on the yield and quality of the cotton.

Major differences exist in cotton planting row spacing, planting density, film mulching width, relative position among drip tape and cotton, as well as cotton-picking mode compared to conventional planting patterns (Wang et al., 2014a). This has different effects on soil temperature, the spatial movement and distribution of water and salt, cotton growth, water consumption, yield and water use efficiency (WUE). However, most studies focused on the conventional planting pattern, rarely involving the machine-harvest cotton planting pattern, and there is a lack of comparative analysis of the typical planting patterns. The objective of this study was to determine the effects of drip tape layouts on cotton yield, quality, water and salt distribution and cotton growth characteristics. Our research group hypothesized that the cotton planting model with one film, three drip tapes and six rows is better than other planting models in terms of soil hydrothermal conditions, agronomic traits, yield and quality of cotton. Therefore, in this study, we performed field experiments were to study the effects of different drip tapes layout patterns on soil water and salt transport, and distribution characteristics, and also quantified the differences in cotton yield, quality, and WUE under different drip tape arrangements. According to the current field cotton production conditions, combined with the local light and heat, soil, machinery, and other conditions, it is of great significance to explore a reasonable planting mode of drip irrigation under mulch film for rational control of soil water and salt distribution in cotton fields. It is also important to further improve water-saving technology, improve water and fertilizer utilization efficiency, and control soil salinity near crop roots during the growing period for the sustainable development of oasis agriculture.

## Materials and Methods

### Experimental site

**Figure 1:**
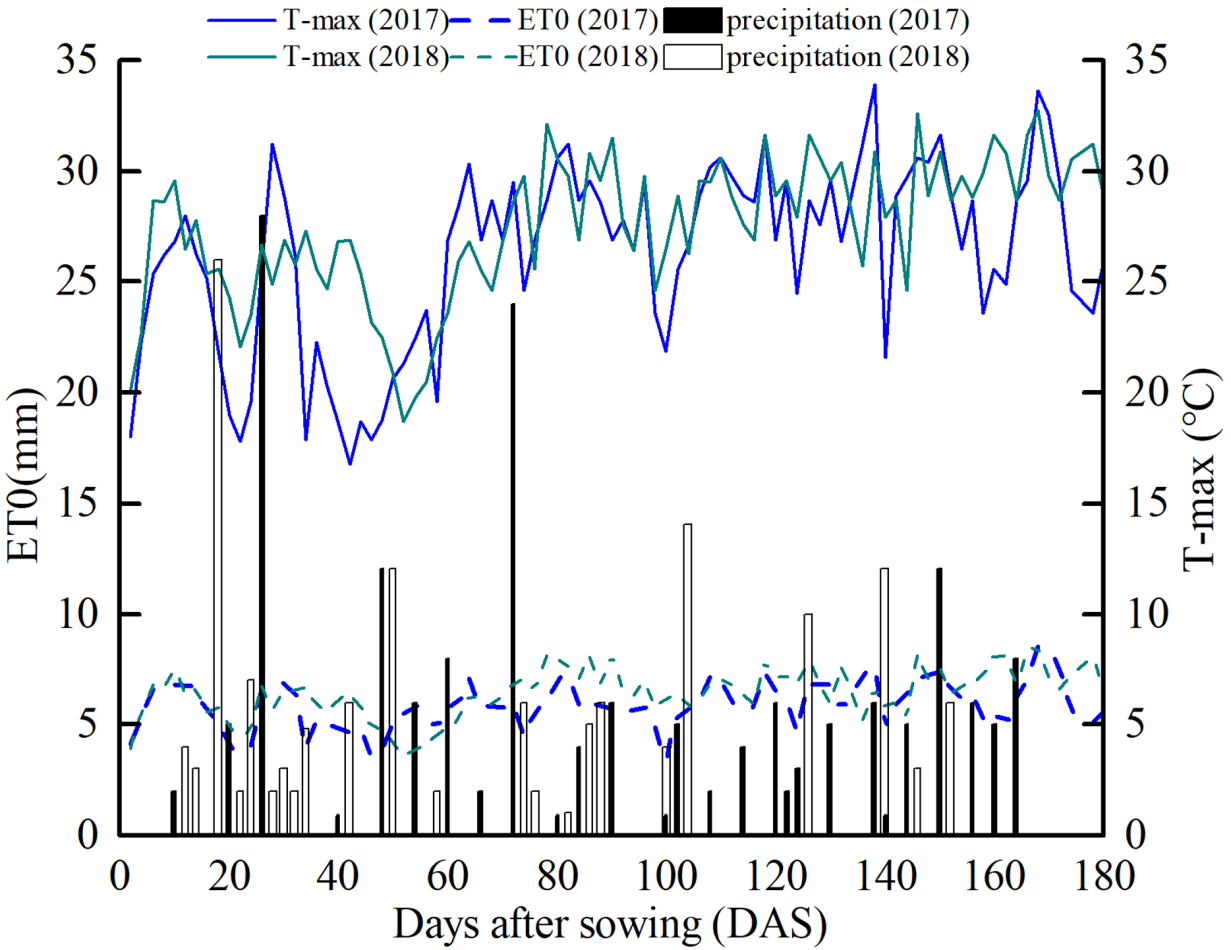
Meteorological data map of cotton growing season at the experimental site in 2017 and 2018. T-max (the maximum air temperature, indicated by the curves), daily precipitation (indicated by the bars), and ET0 (daily reference evapotranspiration, indicated by the dashed curve) during the cotton growing season at the experimental site in 2017 and 2018.

The experiment was conducted at the Key Laboratory of Modern Water-Saving Irrigation of the Xinjiang Production and Construction Corps (85°59′ E, 44°19′ N, altitude 412 m) from April 2017 to November 2018 at Shihezi University in Xinjiang, China. The region experiences a typical arid continental climate, with an average annual rainfall of 210 mm, air temperature of 7.2 °C, sunshine duration of 2,865 h and frost-free period lasting 171 days. The average accumulated temperatures above 10 °C and 15 °C are 3,463 °C and 2,960 °C, respectively. Changes in precipitation, daily reference evapotranspiration (ET0), and maximum atmospheric temperature in the cotton-growing season (from April to November) in 2017 and 2018 are presented in Fig. 1. The total rainfall values in the cotton-growing season in 2017 and 2018 were 166 mm and 153.8 mm, respectively. Air temperature, precipitation, wind speed, and other meteorological data were recorded by an automatic weather station.

The experimental area was 0.06 ha. The regional ground-water table remained at a depth of 8 m. The local soil was medium loam with a pH of 7.83, a mean field water holding capacity of 19.5%, and mean permanent wilting point of 9.8%. In the surface soil (0–20 cm in depth), the amounts of soil organic matter, total nitrogen (N), phosphate (P), and available potassium (K) were 19, 0.08, 0.94, and 0.42 g kg^–1^, respectively. The cotton variety ‘Nong feng 133’ is suitable for dense planting, good ventilation, light transmission among populations, early maturity, and high yield. The soil physical conditions (*e.g*., dry bulk density and field water holding capacity) of 0–100 cm soil layers in the test area are shown in Table 1.

**Table 1:** Soil physical properties in the study area in 2017 and 2018.

Years	Soil depth/cm	0–10	10–20	20–30	30–40	40–50	50–60	60–70	70–100	Mean value
2017	Dry bulk density (g/cm^3^)	1.45	1.46	1.52	1.53	1.54	1.59	1.67	1.63	1.55
	Soil moisture content (%)	20.88	20.49	21.24	20.65	20.37	20.87	21.51	22.89	21.11
2018	Dry bulk density (g/cm^3^)	1.48	1.36	1.34	1.36	1.40	1.42	1.51	1.36	1.40
	Soil moisture content (%)	18.69	19.84	21.36	21.54	21.79	22.00	24.56	22.43	21.53

### Experimental design

**Figure 2:**
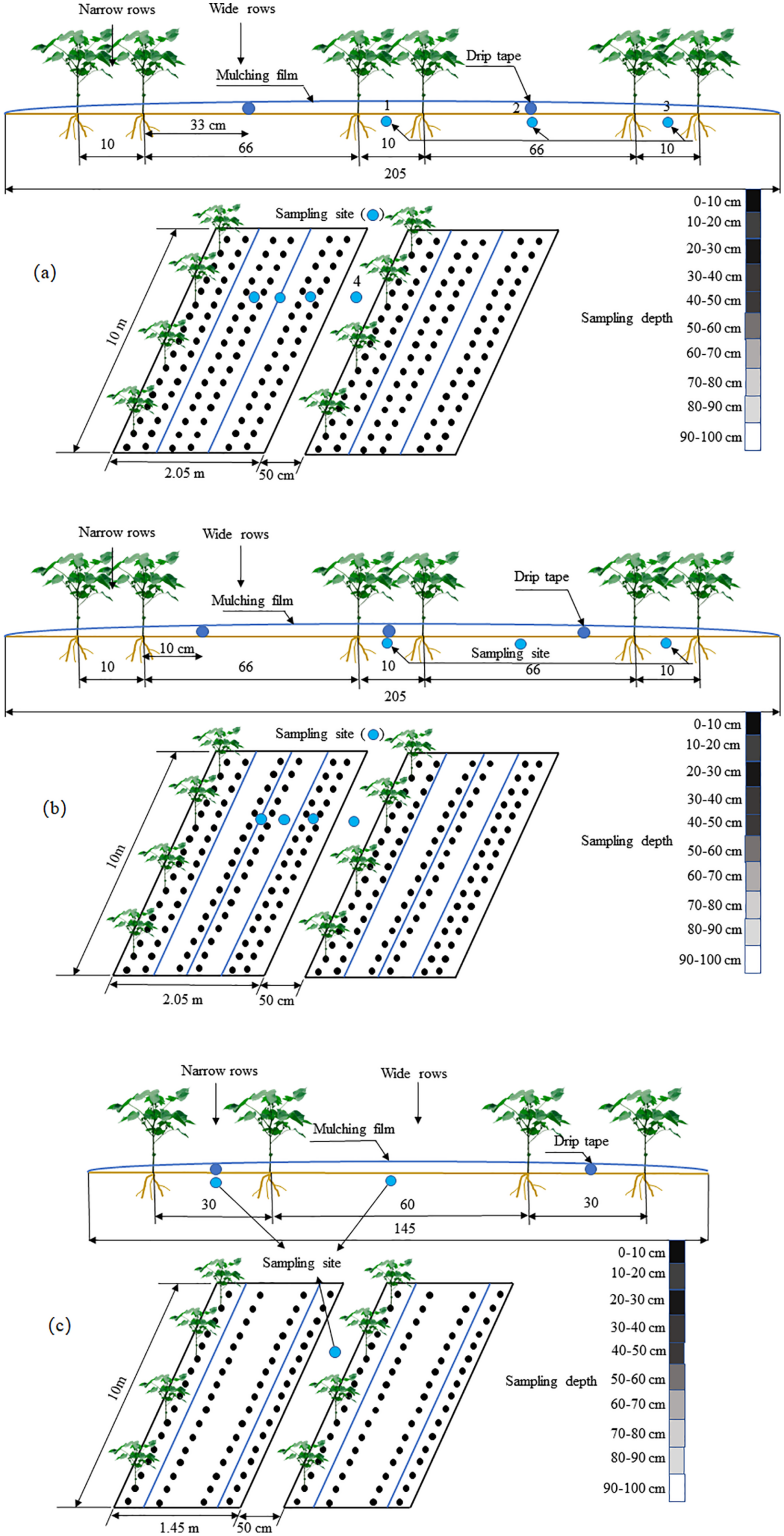
Schematic diagram of different layout patterns of drip tape under the film, and layout of the mulched drip irrigation design used in this study. (A) The cotton planting model with one film, two drip tapes and six rows; (B) the cotton planting model with one film, three drip tapes and six rows; (C) the conventional cotton planting model with one film, two drip tapes and four rows.

The experiment was prepared following a randomized block design with three treatments. T1: the cotton planting model with one film, two drip tapes and six rows (Fig. 2A); the drip tape was arranged in the middle of the wide cotton rows. The planting row space was 10 cm + 66 cm, and the theoretical planting density was 0.26 million plants hm^–2^. T2: the cotton planting model with one film, three drip tapes and six rows (Fig. 2B); one drip tape was arranged in the middle of the narrow cotton row, and the others were arranged at 10 cm away from the inner side of the narrow row of cotton. The planting row space was 10 cm + 66 cm, and the theoretical planting density was 0.26 million plants hm^–2^. T3: the conventional cotton planting model with one film, two drip tapes and four rows (Fig. 2C); the drip tape was arranged in the middle of narrow rows of cotton. The planting spacing was 30 cm + 60 cm + 30 cm, and the theoretical planting density was 0.22 million plants hm^–2^. Each treatment included three repeats, a total of nine test plots. The area of each test plot was 10 m × 6 m, and there were 1 m protection rows between the test plots. Among them, T1 and T2 were machine-harvest cotton planting modes, and T3 was a traditional manual-picking cotton planting mode. The differences between the cotton row spacing and drip tapes of the various planting modes can lead to certain differences in the cotton planting density. For example, the traditional mode has a lower density and the machine-harvest cotton is greater in amount. The specific details of the three layout modes are shown in Fig. 2.

The plant spacing of cotton in the machine harvesting and traditional planting modes was 10 cm and 30 cm, respectively (Fig. 2), and the cotton was sown in a way of “dry sowing and wet out”. The cotton planting dates in 2017 and 2018 were April 20 and April 22, respectively. During the growth period, the drip irrigation method under the film was used to provide the necessary water and nutrients for cotton growth. The drip irrigation capillary was a single-wing labyrinth thin-walled drip tape. The emitter flow rate was 2.6 L h^–1^. The distance between the drippers was 30 cm, and the thickness of the plastic film was (0.008 ± 0.0003) mm. The water supply system in the study area was mainly pressurized by a water pump, and the pressure gauge and regulating valve were installed at the head of the system. The irrigation quota, irrigation volume, irrigation date, irrigation times, and fertilization times of each planting mode were the same during the experiment. The cotton was irrigated 13 times during the whole growth period. The irrigation quota was 450 mm; the N fertilizer was applied at 225 kg hm^–2^ and the P fertilizer at 140 kg hm^–2^ during the whole growth period. Irrigation was carried out by drip irrigation under the mulch. The irrigation interval was 7–10 days. The squaring period occurred four times, the flowering period occurred four times, the bolling period occurred three times, and maturity occurred two times. The fertilizer was dissolved in the water for fertilization while irrigating. The details of irrigation and fertilization management during the cotton growth period are shown in Table 2.

**Table 2:** Irrigation schedule during the cotton growing season in 2017 and 2018.

Growth stage*	2017		2018	
	Irrigation date	Irrigation quota(mm)	Irrigation date	Irrigation quota(mm)
Squaring	April 22, 2017	20	April 24, 2018	20
	June 16, 2017	25	June 9, 2018	25
	June 24, 2017	25	June 16, 2018	25
	June 30, 2017	25	June 23, 2018	25
Flowering	July 7, 2017	50	July 1, 2018	50
July 14, 2017	50	July 9, 2018	50	
July 21, 2017	50	July 16, 2018	50	
July 28, 2017	50	July 23, 2018	50	
Bolling	August 4, 2017	35	July 30, 2018	35
August 11, 2017	35	August 6, 2018	35	
August 18, 2017	35	August 13, 2018	35	
Maturity	August 25, 2017	25	August 20, 2018	25
September 1, 2017	25	August 27, 2018	25	
Total quota (mm)	450	450		

**Note:**

* Squaring indicates that a full canopy surface area develops; flowering indicates the beginning of flowering; bolling indicates boll development; boll opening indicates the beginning of boll bursting; and maturity indicates that over 90% of bolls open. Definitions of cotton phonological stages are from Munger et al. (1998).

### Sampling and field measurements

#### Soil temperature

The soil temperature was measured at 5, 10, 15, 20, and 25 cm depths in each plot using geothermometers (Hongxing Instruments Factory, Hebei Province, China). Each set of geothermometers was inserted between the wide and narrow rows of cotton planting in each plot. In 2017 and 2018, soil temperature was measured at 08:00, 12:00, 14:00, 16:00, 18:00 and 20:00 on the day of seedling (12 days after sowing, DAS), squaring (50 DAS), flowering (65 DAS), bolling (90 DAS), boll opening (120 DAS), and maturity (180 DAS). The automatic weather station in the test station was used to monitor the real-time atmospheric temperature.

#### Soil water storage

In 2017 and 2018, gravimetric soil moisture was measured to a depth of 100 cm at 10 cm intervals on 45, 90, 135, and 180 DAS, respectively. On the measurement day, in T1, T2, and T3 models, three soil cores were auger drilled in each plot to collect the samples from: one below the drip tape, one in the middle of the wide row under the mulch film, and one below the bare soil between films (Fig. 2). Soil samples were collected by ring knife (100 cm^3^) and dried to measure soil moisture and bulk density. Soil samples were taken from each depth and repeated three times. A detailed description of soil sampling and soil moisture measurement was provided in Li et al. (2013). The volumetric water content was calculated by gravimetric water content multiplied to the soil bulk density. Soil water storage at 0–100 cm depth was calculated.

#### Soil salt content

Soil samples were taken from wide row, narrow row and bare land between mulching film at different growth stages of cotton. The soil samples were dried, ground and passed through a 1 mm sieve. Each sample was mixed with 20 g soil according to the ratio of soil and water mass 1:5 and then placed for 2 h after shaken evenly. The conductivity of supernatant was measured by conductivity meter (DDS11-A). The absolute change (ΔS) and relative change (R) in soil salinity were calculated as:

(1) $$S = E{C_{1:5}}^{\hskip -.6pc\prime} - E{C_{1:5}},$$

(2) $$R = {{\Delta S}\over {{\rm{EC}}_{1:5}}} \times 100\% ,$$

where, *EC*_1:5_ and *EC′*_1:5_ (ds m^–1^) represent soil salinity before sowing and after harvest, respectively.

#### Aboveground dry matter, cotton growth, plant height, and leaf area

The aboveground dry matter was measured from 45 DAS and continued at the 45-day intervals after seeding in 2017 and 2018. On each measurement day, the aboveground parts of five cotton plants were collected. The plant samples were dried to a constant weight to measure the biomass. Moreover, the date the plants entered each phenological stage (*e.g*., emergence, squaring, flowering, boll opening, and maturity) was recorded in all plots. The definition of each phenological stage was adopted from Munger et al. (1998). Five representative cotton plants were selected randomly in each plot at the emergence stage. The plant height, leaf length, and leaf width were measured at the 10–15 days intervals after seeding using a tape measure with an accuracy of 1 mm. The leaf area was measured using an empirical coefficient formula (0.75 × leaf length × leaf width) and a handheld leaf area tester (Yaxin-1241) (Wang et al., 2017).

#### Cotton yield and water use efficiency (WUE)

Cotton yield was determined by hand harvesting the center rows (2.05 m × 10 m, 1.45 m × 10 m) in each plot and convert actual yield into total yield per hectare (kg ha^−1^). The water use efficiency (WUE, kg ha^−1^ mm^−1^) was calculated as the ratio between annual cotton yield and total evapotranspiration (ET) over the growing season in each year (Hussain & Aljaloud, 1995). Since the depth of groundwater in the experimental area during the cotton growing season was 8 m, the impact of groundwater replenishment on the water demand of cotton was not considered; The irrigation method was drip irrigation under mulch, which belongs to small quota irrigation, so deep seepage and surface runoff were not considered, the ET could be calculated as:

(3) $$ET = P + I + \Delta SWS,$$

(4) $$WUE = \displaystyle{{\rm Y} \over {{\rm ET}}}$$

where *I* represents irrigation, *P* represents rainfall, and Δ*SWS* represents the difference in soil water storage in the 0–100 cm depth of soil between sowing and harvest. *Y* is the annual cotton yield (kg hm^–2^).

#### Cotton fiber quality

Before harvesting, select a representative area of 4 m^2^ (2 m × 2 m) in each plot to measure the boll weight and lint. The cotton lint sample was sent to the Cotton Quality Supervision, Inspection and Test Center of the Ministry of Agriculture and Rural Affairs to test the quality of cotton fiber with a high-volume instrument (HVI) (Wang et al., 2019).

### Statistics and analysis

The test data were graphed and processed using SPSS 20.0 (IBM SPSS Statistics, USA) and Origin 9.0. Measurement data were analyzed using the one-way analysis of variance (ANOVA) tests. Significantly differences among various treatments are calculated through the least significant difference (LSD) at *P* < 0.05 level.

## Results

### Soil temperature

**Figure 3:**
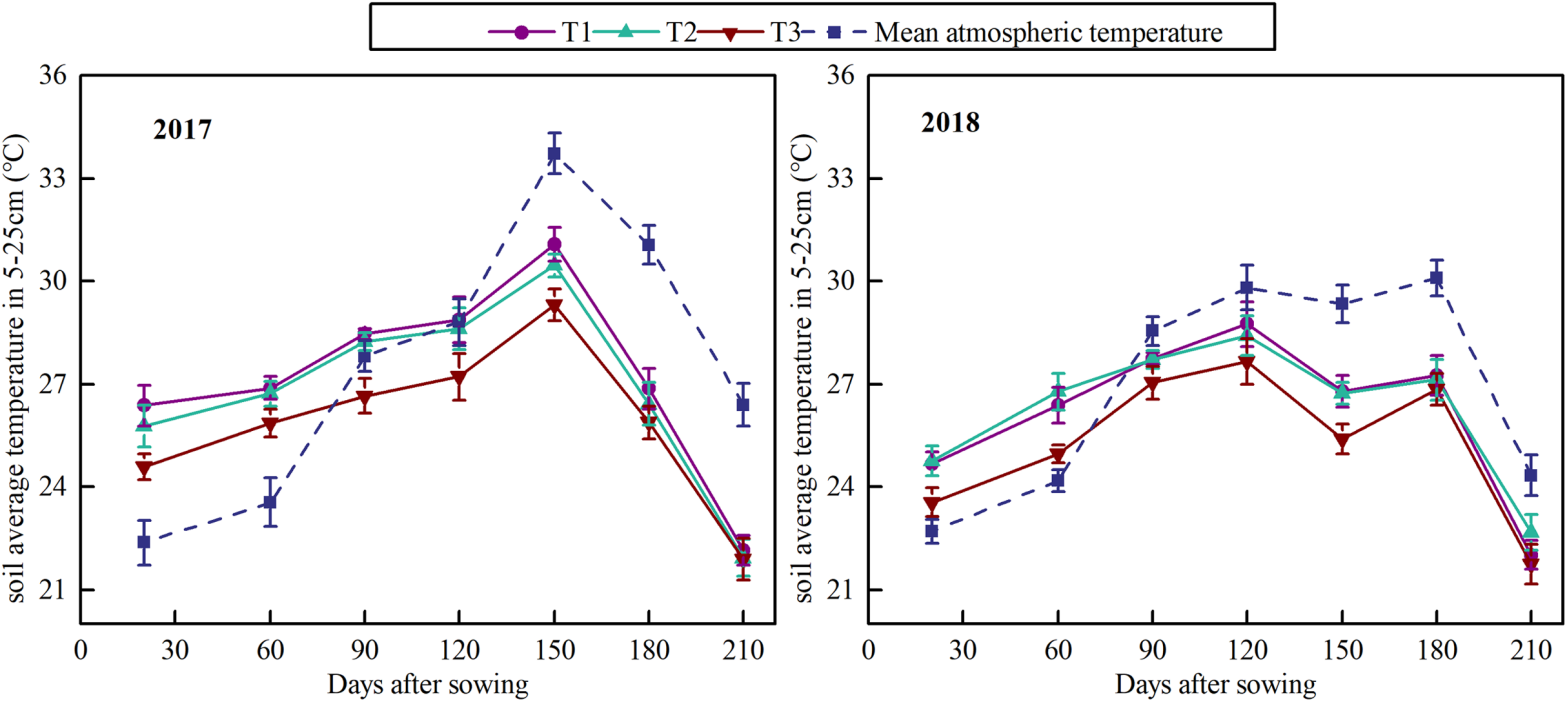
Variation of the mean soil temperature at 5–25 cm with days after sowing across different planting patterns. The error bars in the figure legends represent the standard deviation.

Differences in mean atmospheric temperature and mean temperature of the topsoil (5–25 cm depth) between the plots with different drip tape modes across days after sowing are shown in Fig. 3. At the early cotton growth stage (0–40 DAS), the mean soil temperature values in the T1, T2, and T3 treatments were slightly higher than the mean atmospheric temperature (Fig. 3). Moreover, the atmospheric temperature was lower at the emergence stage. The mulching film increased the surface temperature of the soil in 2017 and 2018. At the medium growth stage (60 DAS), the mean soil temperature values in T1, T2, and T3 treatments were higher than that of the atmosphere (Fig. 3). The temperature difference between T2 and T1 was not significant, but both were higher than that in T3 in 2017. The data in 2018 showed that the mean soil temperature in T2 > T1 > T3 in different drip tape modes. At the late growth stage (180 DAS), the integrity of the plastic film had been destroyed due to natural environmental factors. The mean soil temperature of 5–25 cm soil decreased with the atmospheric temperature decrease, and there were no significant differences among the treatments (Fig. 3). These results showed that wide film provided better warming and heat preservation effect at the initial stage of cotton, and the effect of film mulching width on soil temperature was weakened at later stages of cotton growth (Fig. 3).

### Soil water storage

**Figure 4:**
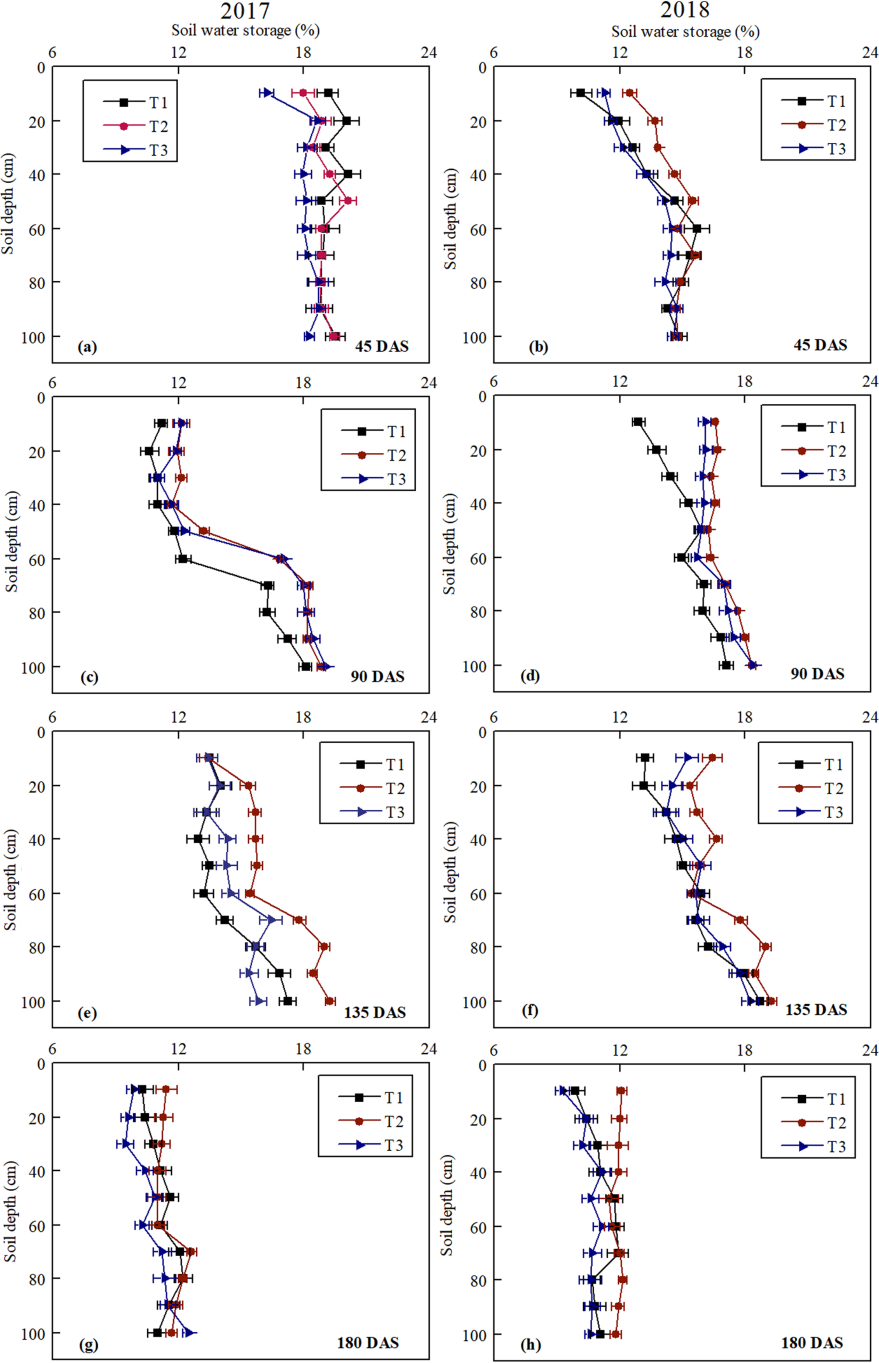
Differences in soil water storage (0–100 cm depth) of plots under different planting modes on different days after sowing (DAS) in 2017 and 2018. T1, T2, and T3 indicate different planting modes. (T1: the cotton planting model with one film, two drip tapes and six rows; T2: the cotton planting model with one film, three drip tapes and six rows; T3: the conventional cotton planting model with one film, two drip tapes and four rows). The error bars in the figure legends represent the standard deviation.

The wetting effects of different drip tape modes at 45 DAS, 90 DAS, 135 DAS, and 180 DAS representing different cotton-growing stages are shown in Fig. 4. At the early growth stage (Figs. 4A, 4B), due to the slow growth of cotton, relatively less rainfall and little surface evaporation, the change in the trends of soil moisture in the T1, T2 and T3 treatments was similar. However, the mean soil moisture at 0–100 cm in T1 and T2 was higher by 6.24%, 5.00% and 2.19%, 7.44% than T3 in 2017 and 2018, respectively. At the middle growth stage (Figs. 4C, 4D), the soil moisture of the surface layer and root layer dramatically changed. Compared to T1 and T3, the mean soil moisture in T2 was higher by 11.59%, 1.28%, and 10.79%, 2.33% in 2017 and 2018, respectively. The mean soil moisture of 0–60 cm soil layer in T2 was higher 2.68% and 2.97% than T3 in 2017 and 2018, respectively. The water holding capacity of the middle and upper part in T2 plow layer was better than that in T3. At the mid-late growth stage (Figs. 4E, 4F), the appropriate soil moisture should maintain 70% of the field water holding capacity to meet the basic water needs of cotton growth when cotton began to open bolls. Comparing the soil moisture content at 0–60 cm (cotton root layer) under the three modes, our results showed that the mean soil moisture values of 0–60 cm soil in T1, T2, and T3 were 13.42%, 15.22%, 13.99% and 14.37%, 15.87%, 15.09% in 2017 and 2018, respectively. The relative field water holding rates were 63%, 71.53%, 65.75%, and 67.54%, 74.27%, 69.41% in 2017 and 2018, respectively. At the late growth stage (Figs. 4G, 4H), irrigation was stopped and the mulching film gradually degraded in the natural environment. The soil moisture values in T1, T2, and T3 were only slightly different. In general, our results showed that T2 was the most effective mode in wetting soil across all cotton-growing stages (Fig. 4).

### Soil salinity

**Figure 5:**
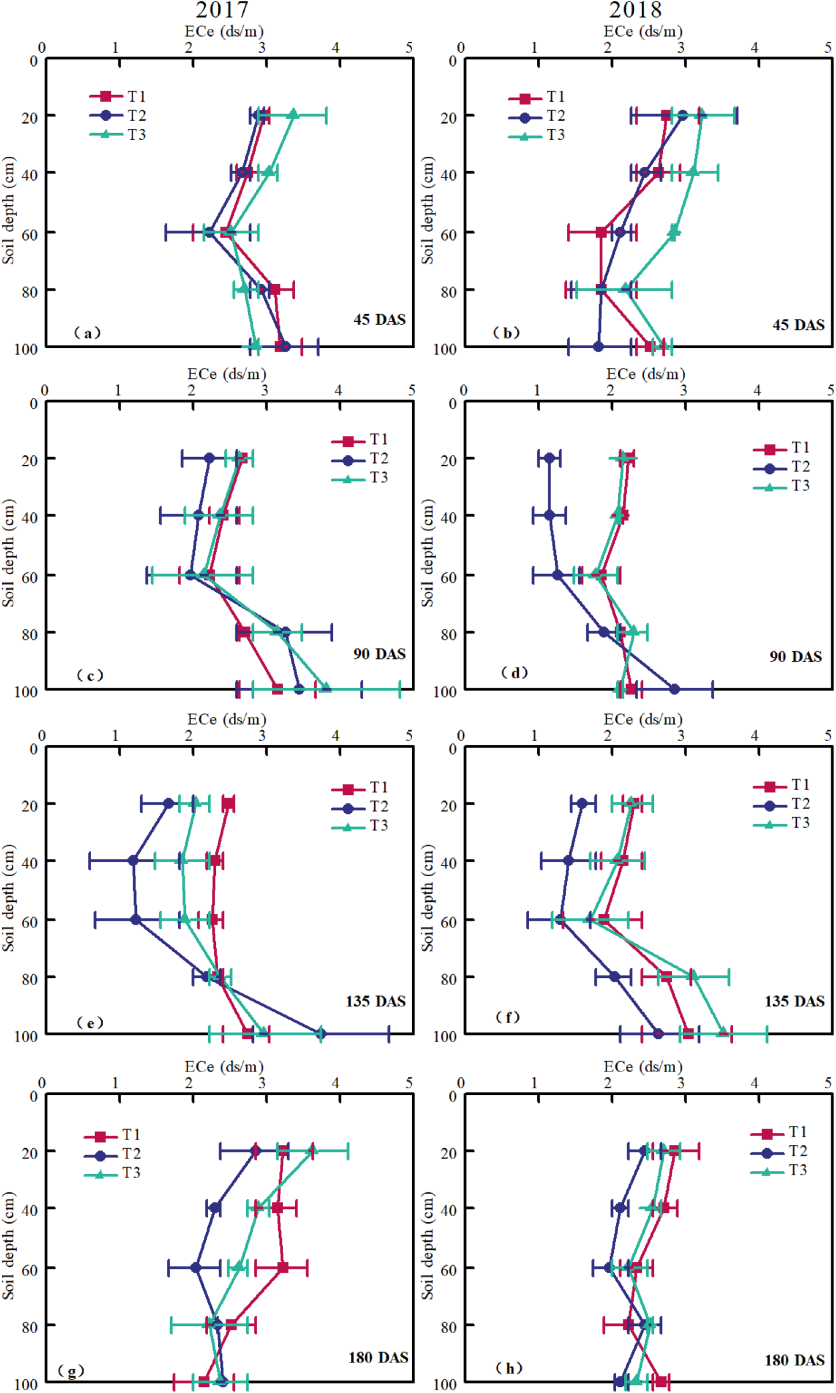
Differences in soil salinity (EC_1:5_) of plots under different planting modes at different soil depths measured on different days after sowing (DAS) in 2017 and 2018. T1, T2, and T3 indicate different planting modes of cotton (T1: the cotton planting model with one film, two drip tapes and six rows; T2: the cotton planting model with one film, three drip tapes and six rows; T3: the conventional cotton planting model with one film, two drip tapes and four rows). The error bars in the figure legends represent the standard deviation.

The desalinizing effects of different planting patterns at 45 DAS, 90 DAS, 135 DAS, and 180 DAS representing different cotton-growing stages are shown in Fig. 5. In general, the total salt content of the soil showed a trend of first decreasing and then increasing within the range of 0–100 cm. At the early cotton growth stage (Figs. 5A, 5B), less irrigation and strong evaporation led to increased salt accumulation in the shallow soil (10–30 cm depth). The salt content first decreased and then increased with the soil layer depth. The salinity in the 0–60 cm soil layer was T3 > T1 > T2 and fluctuated under T1, T2, and T3 in the 60–100 cm soil layer. At the squaring stage (Figs. 5C, 5D), with the increase of irrigation times, the salt moved down with the wetting front, the salt content significantly decreased, especially for soil from 0 to 40 cm depth. At this stage, the salinity in 0–40 cm soil layer was lower than that in the early growth stage of cotton. However, the soil salt contents in T1 and T3 were significantly higher than those in T2 in 0–60 cm soil layer, but the difference was small under T1 and T3. At the boll opening stage (Figs. 5E, 5F), with the increase of irrigation times and irrigation quota, the salt content of shallow soil significantly decreased. The reason is that T1, T2 and T3 are irrigated by drip irrigation under mulch film, and the leaching effect of drip irrigation creates a good salt environment for cotton roots. At this stage, the salinity of the 0–60 cm soil layer was T1 > T3 > T2. At the late cotton growth stage (Figs. 5G, 5H), the soil salinity moved up again due to the strong evaporation and stop of irrigation. At this stage, the soil salinity under different modes of the 0–60 cm soil layer was T1 > T3 > T2.

Table 3 shows the total changes in soil salinity of topsoil measured after cotton harvest compared to the condition right after the sowing. The soil salinity change was significant during the cotton growing season in 2017 and 2018. Soil salinity change rate was the highest in T3, whereas the lowest in T2. Among the three modes, T2 planting mode was more efficient in controlling soil salt accumulation. The accumulation of soil salt was weakened with soil depth, suggesting an upwards movement of salts from the deeper subsoil to surface soil due to evaporation as the primary factor that induced soil salinization. In conclusion, the desalination zone was formed near the root layer of cotton in T2, and the salt accumulation zone was formed at the edge of the wetting front, which was beneficial to cotton growth.

**Table 3:** The absolute (ΔS) and relative (R) change in soil salinity of topsoil measured after cotton harvest compared with that measured right after sowing.

Treatment	Soil salinity change	2017				2018			
		0–10 cm	10–20 cm	20–30 cm	30–40 cm	0–10 cm	10–20 cm	20–30 cm	30–40 cm
T1	ΔS (ds m^–1^)	0.406	0.239	0.211	0.156	0.383	0.256	0.217	0.172
R (%)	37.9	16.6	12.1	5.5	32.9	17.6	12.1	7.1	
T2	ΔS (ds m^–1^)	0.389	0.211	0.156	0.128	0.289	0.217	0.256	0.128
R (%)	33.3	13.3	6.0	2.7	23.3	13.3	16.0	2.7	
T3	ΔS (ds m^–1^)	0.450	0.239	0.238	0.183	0.428	0.306	0.266	0.217
R (%)	43.5	16.7	15.42	8.3	37.9	23.3	17.64	11.1	

**Note:**

T1, T2 and T3 indicated different planting modes of cotton (T1: the cotton planting model with one film, two drip tapes and six rows; T2: the cotton planting model with one film, three drip tapes and six rows; T3: the conventional cotton planting model with one film, two drip tapes and four rows).

### Growth characteristics and quality of cotton

Table 4 shows the effects of different drip tape modes on agronomic characteristics and quality of cotton. The cotton plant height, leaf area index, and leaf dry matter mass under T2 were significantly higher than T1 and T3. Cotton stem diameter expressed no significant differences in T1 and T2 but it was significantly higher than that in T3. The suitable plant height of cotton machine picking is 70–75 cm. Cotton plant height in T2 was more conducive to machine harvesting than in T1 and T3 (Table 4). The cotton quality analysis showed no significant differences in elongation at break among T1, T2, and T3. The mean length of the upper half of cotton, length uniformity index, micronaire value, breaking ratio length, and lint percentage showed significant differences where T2 was significantly higher than T1 and T3. There were no significant differences in other quality indicators in T1 and T3 except for the lint. Compared to T1 and T3, the cotton lint in T2 was 14.9%, 7.7%, and 11.0%, 3.0% higher in 2017 and 2018, respectively. Overall, the cotton agronomic characteristics and quality in T2 were relatively better than those in T1 and T3.

**Table 4:** Analysis of agronomic characters and quality of cotton under different drip tape arrangement patterns.

		Cotton growth characteristics					Cotton quality					
		Plant height (cm)	Plant stem (mm)	Leaf area index	Dry matter (g)		Upper half mean length (m)	Length uniformity index (%)	Micro-naire	Breaking strength (Cn/tex)	Elongation (%)	Lint (%)
2017	T1	61.2b	10.5ab	2.1b	65.8ab		27.9b	84.2b	4.8b	26.5b	6.9a	36.4c
	T2	71.8a	11.4a	3.8a	71.6a		33.1a	87.7a	5.16a	31.6a	7.1a	41.8a
	T3	49.9c	9.4c	2.3b	61.4c		28.4b	83.5b	4.8b	27.2b	7.0a	38.8b
2018	T1	64.1b	11.5a	2.4b	66.7ab		28.4b	84.3c	4.7b	25.3b	6.8a	37.4c
	T2	75.9a	11.8a	4.1a	72.7a		32.2a	88.3a	5.06a	30.8a	7.0a	41.5a
	T3	53.6c	9.6b	2.6b	54.4c		28.5b	82.7c	4.9ab	26.4b	6.9a	40.3ab

**Note:**

Different superscript letters (a, b, and c) indicate significant differences between treatments in a year (*p* ≤ 0.05). T1, T2 and T3 indicated different planting modes of cotton (T1: the cotton planting model with one film, two drip tapes and six rows; T2: the cotton planting model with one film, three drip tapes and six rows; T3: the conventional cotton planting model with one film, two drip tapes and four rows).

### Cotton yield and water use efficiency (WUE)

**Figure 6:**
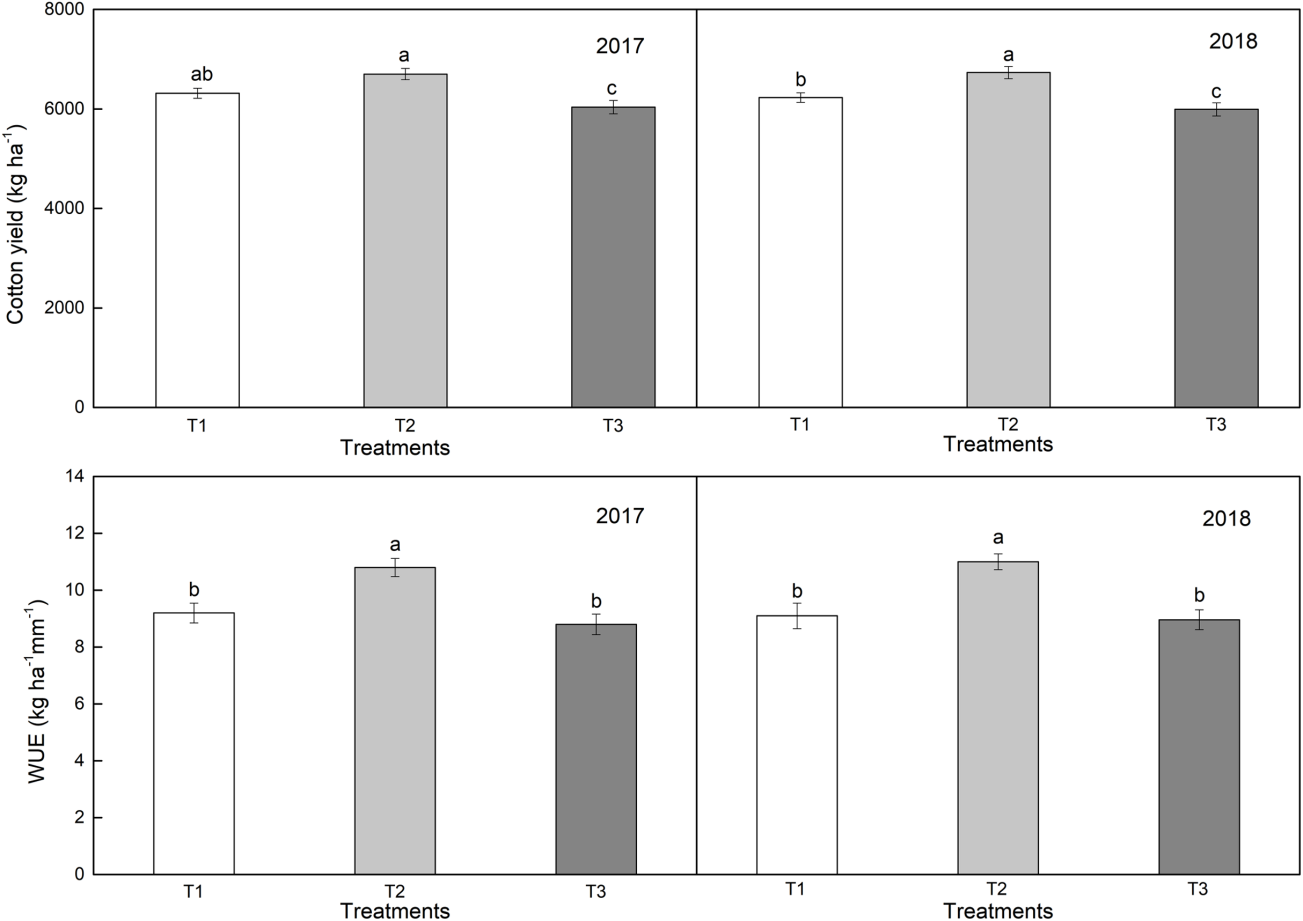
Effects of different planting patterns on cotton yield and water use efficiency (WUE) in 2017 and 2018. T1, T2, and T3 represent different planting modes of cotton. Different superscript letters (a, b, and c) indicate significant differences between treatments in each year (*p* ≤ 0.05) (T1: the cotton planting model with one film, two drip tapes and six rows; T2: the cotton planting model with one film, three drip tapes and six rows; T3: the conventional cotton planting model with one film, two drip tapes and four rows).

Among different drip type modes, T2 performed the best in terms of enhancing the cotton yield in both years (*i.e*., 6,701 kg ha^–1^ in 2017 and 6,730 kg ha^–1^ in 2018) (Figs. 6A, 6B). The mean increases in cotton yield in T2 relative to T1 and T3 in 2017 and 2018 were 6.15% and 11.0% and 8.1% and 12.3%, respectively. In general, cotton yield under different planting patterns was T2 > T1 > T3, and there were significant differences between T2 and T3.

The WUE in T2 was the highest (Figs. 6C, 6D), which was significantly higher than that in T1 and T3, which were 10.8 kg/ha/mm and 11.0 kg/ha/mm in 2017 and 2018, respectively. Compared to T1 and T3, there was an increase of 17.4%, 22.7%, and 20.9%, 22.8% in T2 WUE in 2017 and 2018, respectively, suggesting that the drip tape mode in T2 can be more eﬀective in improving WUE. There were no significant differences between T1 and T3. Therefore, T2 planting mode was likely more effective in improving yield and WUE. The drip tape mode in T2 is recommended for machine-harvest cotton planting in arid areas of Xinjiang, China.

## Discussion

Differences in the mean temperature of the topsoil (5–25 cm depth) between the plots and atmospheric temperature under the three drip tape modes under mulch are compared in Fig. 3. Plastic film mulching has been used to improve soil temperature and reduce soil evaporation since 1975 (Kasirajan & Ngouajio, 2012). Other studies have indicated that the soil temperature significantly increases with the increase in film mulching width (Wang et al., 2018b). The wide film can improve radiation capture and soil temperatures and increase available soil water, and thus increasing crop yield (Steinmetz et al., 2016). This is consistent with our observations of soil temperature, and the temperature difference between T2 and T1 was not significant, but both were higher than that of T3 (Fig. 3). Moreover, in our study, the warming effect was more evident in the early stages of cotton growth (particularly from seeding to flowering). Then, the warming effect gradually weakened with the growth of cotton, which was consistent with the findings of Kelley & Langston (2006). Different drip tape modes have different effects on soil hydrothermal conditions (Qi et al., 2016; Liu et al., 2013). There were significant differences in soil moisture distribution under different cotton planting patterns (Li et al., 2013; Qin et al., 2014; Qin, Hu & Oenema, 2015). A similar situation was found in our study. Under the same planting conditions, the soil moisture in T2 was higher than that in T1, which can provide moderate water for cotton roots and effectively reduce water stress for cotton. Liu et al. (2012a) showed that the soil moisture content (SMC) of the narrow cotton rows in the single-drip irrigation zone is lower than that in the double-drip irrigation zone, which is similar to our study finding. In our study, the drip tape was placed between the wide rows of cotton in T1. The soil moisture was mainly distributed between the wide rows of cotton, and the soil moisture around the roots of the narrow rows of cotton was relatively less. The drip tape was arranged between the narrow rows of cotton in T3, and the areas with higher soil moisture were distributed between the narrow rows of cotton. Under the machine-harvest mode of super wide film, the planting density and water consumption of cotton are moderate, and the distribution of soil water in the cotton root zone is the most favorable for cotton growth, while the WUE is the highest (Ning, Zuo & Shi, 2013; Liu et al., 2012a). These findings are consistent with our conclusions of soil moisture. In our study, the soil moisture of the 0–60 cm layer in T2 was higher than that in T3, indicating that the water holding capacity of the middle and upper plow layer in T2 was better than that in T3.

Drip irrigation under mulch is small-scale irrigation, which can effectively maintain soil moisture and evenly drive out salt (Akça et al., 2020). In the process of drip irrigation, the salt in the soil has a directional distribution (Wang et al., 2014b). Using drip irrigation, the wetting front of the double pipe arrangement forms intersection in wide rows, and the salt is leached out of the main root layer with water. Therefore, the effect of salt control is better than that of the single pipe (Zhao et al., 2015; Wang et al., 2016). A similar situation was found in our study. The drip tapes in T2 and T3 were arranged closer to the cotton plants so that the cotton root zone was in the center of the wetting body, and the salinity of the cotton main root zone was driven out of the root layer during the water redistribution process. T1 drip irrigation zone was far away from the cotton plant, and the edge of the wetting peak moved to the narrow row of cotton, which caused salt stress for the root zone of cotton. At the late growth stage (Figs. 5G, 5H) when strong evaporation likely induced soil salinization (Marcos et al., 2018; Yang et al., 2011), and mulched drip irrigation washed the soil salt out from the root zone (0–60 cm depth). However, due to the small rate of discharge of mulched drip irrigation, soil salt was difficult to flush into the groundwater (8 m depth) and accumulated beneath the root zone (Zhai, Yang & Wu, 2016; Wang, Fan & Guo, 2019). This finding was consistent with our study. In our drip tape modes, the mean salt content of 0–60 cm soil layer in T2 was significantly lower than that in T1 and T3 under three drip tape arrangements (Fig. 5). In general, the T2 drip tape layout mode can provide suitable water and heat for the growth of cotton during the growth period and create a desalinated soil environment for the cotton root zone, which is conducive to cotton growth. These features make it an appealing management practice, especially in arid and semi-arid regions.

Zhao, Tian & Ma (2003) indicated that the micronaire value of cotton fiber increased with the increase of planting density; In our study, there were significant differences in the mean length of the upper half of cotton, length uniformity index, micronaire value, breaking ratio length, and lint percentage in the T1, T2 and T3 models, which showed that the T2 model was significantly higher than T1 and T3. There were no significant differences in other quality indicators in the T1 and T3 treatments except for the lint. Compared to T1 and T3, the cotton lint in T2 was 14.9% and 7.7% and 11.0% and 3.0% higher in 2017 and 2018, respectively (Table 4). Crop leaf area index, interception of photosynthetically active radiation (PAR), and accumulation of aboveground photosynthetic matter were positively correlated with planting density (Buxton et al., 1977). Planting density significantly affected the structure and function of crop canopy, and reasonable planting mode was conducive to the formation of reasonable canopy structure, interception of more PAR, improvement of light energy utilization rate, and realization of high yield and quality (Dai et al., 2015). The single boll weight is significantly decreased with the decrease of row spacing, and the total bolls per unit area are increased under the machine-harvested pattern (Cui et al., 2018). The fiber quality of cotton had no significant effect, but the increase of row spacing was conducive to increase the defoliation rate (Wang, Zhang & Chen, 2004; Baker, 1976). The agronomic characters and quality of T2 treatment were better than T1 and T3. The reason was that the distribution of water, fertilizer and salt in the root zone of the T2 drip irrigation belt layout model was beneficial to cotton growth and improved quality. Under the T2 mode, the height of cotton plant is suitable, the quality of seed cotton is good, and it is conducive to machine harvesting.

Drip tape arrangements can affect cotton growth, which will be reflected in the cotton yield and WUE in each treatment (Siebert, Stewart & Leonard, 2006; Liu et al., 2012a). Deng et al. (2002) compared the cotton planting patterns of 58 cm + 18 cm, 55 cm + 20 cm and 66 cm + 10 cm, and indicated that cotton yield was higher under the row spacing of 66 cm + 10 cm, which was similar to our observations of cotton yield (Fig. 6). In addition, Xu et al. (2017) indicated that the number of bolls per plant, lint percentage, boll weight, and yield in the 72 cm + 4 cm model were lower than those in the 66 cm + 10 cm model. In our observations, the mean increases in cotton yield under T2 model relative to T1 and T3 in 2017 and 2018 were 6.15% and 11.0% and 8.1% and 12.3%, respectively. In general, cotton yield under different drip tape modes was T2 > T1 > T3, and there were significant differences between T2 and T1, T3 (Fig. 6).

The planting density in T1 and T2 was the same, but the location of drip tape was different, which can lead to soil water and salt transport differences (Yu et al., 2011). WUE depends on cotton yield and water consumption, which are closely related to planting patterns (Ibragimov et al., 2007; Su, Abudu & Hudan, 2011). In the T2 and T3 modes, the drip tapes were arranged beside the cotton plant, so that the cotton root zone was in the center of the wet body, and the salt in the main root zone was driven out of the root layer during the water redistribution. In the T1 mode, the drip tape was far away from the cotton plant, and the edge of wetting peak was close to the narrow row of cotton, which made the cotton root zone subject to salt stress. The distribution of water and salt in soil had a significant effect on the growth of the cotton. Ning, Zuo & Shi (2013) examined the distribution of soil water and salt under three pipe modes, and six rows mode was the most favorable for cotton absorption and utilization, which was consistent with our cotton yield and WUE results. In our study, under T2, water and salt distributions in the cotton root zone were more conducive to cotton growth. Soil heat preservation, warming, and moisture retention effect were better. The cotton plant was large, fruit branches and fruit nodes were more, which can provide higher yield and WUE (Figs. 6C, 6D). In addition, Li et al. (2018) concluded that the soil WUE of one film three pipe six lines was better than one film two pipe six lines and one film three pipe five lines. A similar situation was found in our study. The WUE in T2 was the highest (Figs. 6C, 6D), which was significantly higher than that in T1 and T3, which were 10.8 kg/ha/mm and 11.0 kg/ha/mm in 2017 and 2018, respectively. Compared to T1 and T3, T2 provided an increase of 17.4%, 22.7%, and 20.9%, 22.8% in WUE in 2017 and 2018, respectively, which also suggests that T2 mode can be more effective in improving WUE (Fig. 6).

## Conclusions

This study evaluated the effects of different drip tape modes of drip irrigation on soil hydrothermal conditions, salt accumulation, cotton growth, and WUE under mulching film over 2 years in an oasis agroecosystem in Xinjiang, northwest China. We came to the following conclusions. Among the three modes, the water holding capacity of the middle and upper parts of the tillage layer in the cotton planting model with one film, three drip tapes and six rows was the best. In this mode, the main root layer of the cotton formed a desalination zone, which was more effective in controlling soil salt accumulation. The cotton plant height, leaf area index, and leaf dry matter mass were significantly higher than in the other two modes. The cotton planting model with one film, three drip tapes and six rows treatment was more effective in improving the yield and WUE. Considering water conservation and improving cotton yield, the cotton planting model with one film, three drip tapes and six rows can be recommended for machine-harvest cotton planting. This is important for promoting cotton growth and yield, improving labor productivity and the quality of machine-harvest cotton, and ensuring healthy and sustainable development of regional cotton-production in Xinjiang, China.

## Supplemental Information

10.7717/peerj.12004/supp-1 Supplemental Information 1 Soil moisture, soil conductivity, cotton yield, water use efficiency and meteorological data of different treatments in 2017 and 2018.

10.7717/peerj.12004/supp-2 Supplemental Information 2 Average soil temperature, soil moisture content, soil salt content, agronomic and quality of cotton, cotton yield and WUE of different treatments in 2017 and 2018.

## Additional Information and Declarations

### Data Availability

The following information was supplied regarding data availability: Raw data are available in the Supplemental Files.
